# Laucysteinamide A, a Hybrid PKS/NRPS Metabolite from a Saipan Cyanobacterium, cf. *Caldora penicillata*

**DOI:** 10.3390/md15040121

**Published:** 2017-04-14

**Authors:** Chen Zhang, C. Benjamin Naman, Niclas Engene, William H. Gerwick

**Affiliations:** 1 Department of Nanoengineering, University of California, San Diego, La Jolla, CA 92093, USA; chz023@ucsd.edu; 2 Center for Marine Biotechnology and Biomedicine, Scripps Institution of Oceanography, University of California, San Diego, La Jolla, CA 92093, USA; bnaman@ucsd.edu; 3 Department of Biological Sciences, Florida International University, Miami, FL 33199, USA; nengene@fiu.edu; 4 Skaggs School of Pharmacy and Pharmaceutical Sciences, University of California, San Diego, La Jolla, CA 92093, USA

**Keywords:** laucysteinamide A, cyanobacteria, blue-green alga, thiazoline alkaloid, cytotoxicity

## Abstract

A bioactivity guided study of a cf. *Caldora penicillata* species, collected during a 2013 expedition to the Pacific island of Saipan, Northern Mariana Islands (a commonwealth of the USA), led to the isolation of a new thiazoline-containing alkaloid, laucysteinamide A (**1**). Laucysteinamide A is a new monomeric analogue of the marine cyanobacterial metabolite, somocystinamide A (**2**), a disulfide-bonded dimeric compound that was isolated previously from a Fijian marine cyanobacterium. The structure and absolute configuration of laucysteinamide A (**1**) was determined by a detailed analysis of its NMR, MS, and CD spectra. In addition, the highly bioactive lipid, curacin D (**3**), was also found to be present in this cyanobacterial extract. The latter compound was responsible for the potent cytotoxicity of this extract to H-460 human non-small cell lung cancer cells in vitro.

## 1. Introduction

Field collections of Pacific tropical marine cyanobacteria have been a prolific source for a wide range of novel bioactive marine natural products [1]. Whereas the Northern Mariana Islands have not previously been identified as “hotspots” of marine biodiversity, their coral reef habitats have been a source of chemically-prolific strains of marine cyanobacteria. For example, obyanamide, a high nM LC_50_ human KB cancer cell cytotoxic agent, was isolated from a collection of the marine cyanobacterium *Lyngbya confervoides* from Saipan, a US territory [2]. The relatively unexplored marine biodiversity of this region is thus an exciting resource for screening for new biologically active natural products.

Such screening efforts are especially relevant to the discovery of anticancer lead compounds, as many of our current Food and Drug Administration (FDA) approved agents in this therapeutic class are derived from, or have been patterned after, natural products. Examples include the vinca alkaloids, taxanes, dolastatins (a lead for auristatin E), and halichondrin B (lead for eribulin) [3], as well as a number of preclinical leads such as curacin A, discodermolide and the ixabepilone [4]. Common to all of these agents is their targeting and disruption of the pivotal function of microtubules within cancer cells, leading to apoptotic cell death.

**Figure 1:**
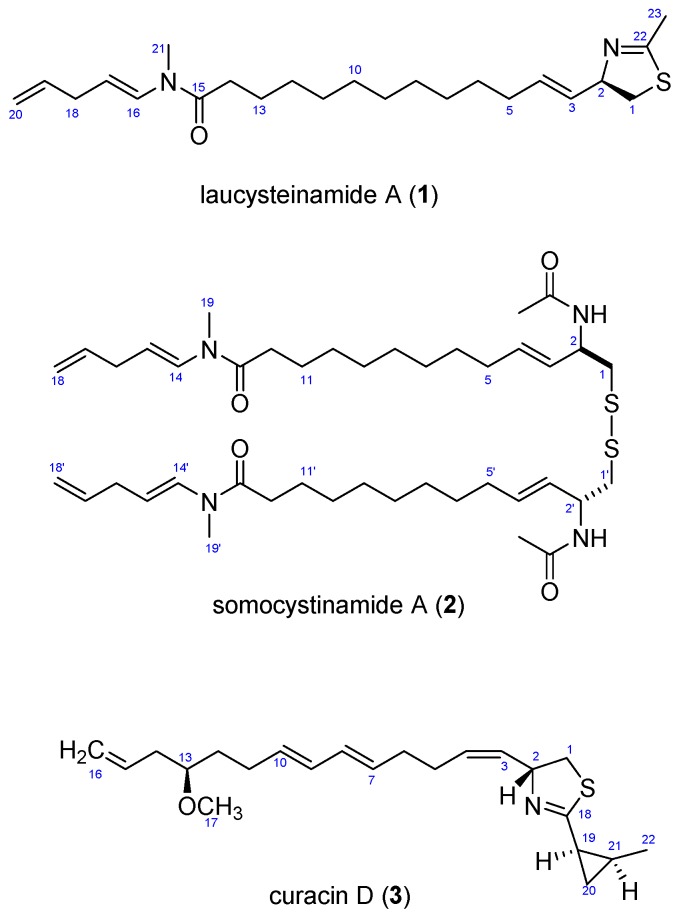
Structures of Compounds (**1**–**3**).

**Figure 2:**
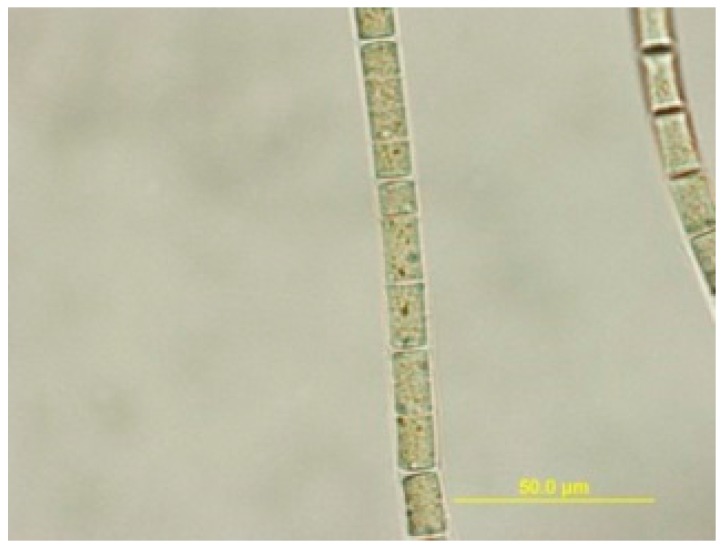
Microscopic image of filaments of the cf. *Caldora penicillata* species (100×).

In 2013, we surveyed the natural populations of marine cyanobacteria from Saipan and made collections of those present in sufficient biomass to support ensuing chemical and pharmacological investigations. One such shallow water (1–2 m) collection, obtained from Lau Lau Bay, comprised several centimeter long tufts of a pinkish-purple colored filamentous cyanobacterium. These were found growing distinctively from the tops of stipes of the brown alga *Turbinaria* sp. Subsequently, the collected biomass was extracted in the laboratory and subjected to a combination of bioassay and Nuclear Magnetic Resonances (NMR) guided isolation efforts. We describe here the isolation, structure elucidation, and biological activity of laucysteinamide A (**1**) (Figure 1), a novel hybrid PKS/NRPS (2-methyl-4-thiazolinyl) cytotoxic compound from this Saipan cyanobacterial collection. A second and highly bioactive metabolite of this collection was the previously described compound curacin D (**3**), a potent inhibitor of microtubule assembly [5]. Laucysteinamide A is structurally related to somocystinamide A (**2**), a neurotoxic and cytotoxic compound (IC_50_ = 3 nM) previously isolated from a mixed assemblage of Fijian marine cyanobacteria [6]. In the course of these studies, the source organism was examined in detail by light microscopy and was found to correspond to the recently described species *Caldora penicillata* (Figure 2) [7].

## 2. Results and Discussion

**Figure 3:**
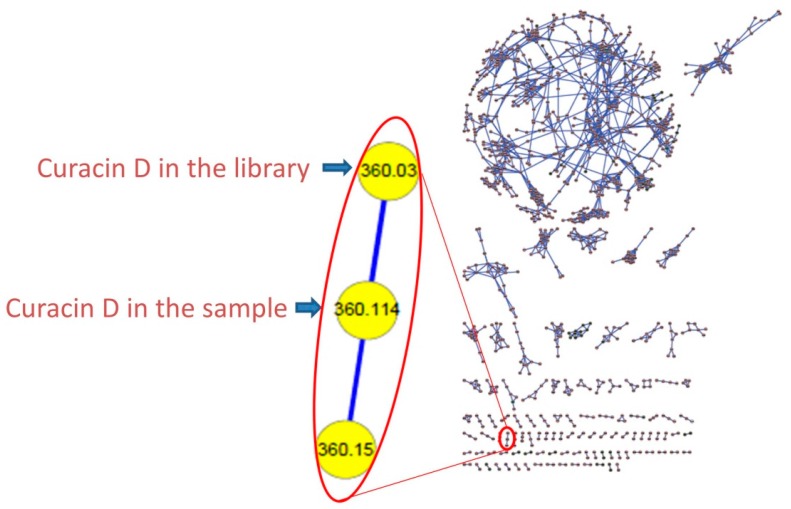
Representative Molecular Network. (**Left**) the expanded cluster of curacin D includes one peak in the LC-MS/MS of the present extract along with the node incorporated from a pure compound library; (**Right**) the entire molecular network of the crude extract, fractions C–J, and the pure compound library. The circular cluster represents structurally similar molecular families from either crude extract or vacuum liquid chromatography (VLC) fractions that share similar MS/MS fragmentation patterns [8].

SPL-3Feb13-2, a cf. *Caldora penicillata* collected at Lau Lau Bay in Saipan, was repeatedly extracted with a 2:1 mixture of dichloromethane (DCM) and methanol (MeOH) to afford a total of 6.73 g of extract. Two lipophilic fractions obtained from vacuum liquid chromatography of this extract (fraction C: 20% ethyl acetate (ETAC)/hexanes *v*/*v*; fraction D: 40% ETAC/hexanes *v*/*v*) possessed interesting ^1^H-NMR features, such as *J*-coupled peaks in the alkenyl and typical peptidyl alpha proton regions. Moreover, they demonstrated strong toxicity in the brine shrimp model (100% toxicity at 3 μg/mL), and thus were selected for further investigation. These two combined fractions (36.5 mg) were repeatedly chromatographed by normal phase High Performance Liquid Chromatography (HPLC) to afford two compounds; 3.6 mg (0.05% extraction yield *w*/*w* dry) of compound **1** as an optically active oil ${\left[\alpha \right]}_{D}^{26}$ = +17.1° (*c* = 0.86, CHCl_3_), and 2.7 mg (0.04% extraction yield *w*/*w* dry) of compound **3**. Dereplication using MS/MS based molecular networking [8] (Figure 3) and ^1^H-NMR [5] indicated that compound **3** was the known cyanobacterial metabolite, curacin D. However, compound **1**, assigned here the trivial name laucysteinamide A, had MS and NMR features unlike any known compound, and hence its structural and biological properties were investigated as reported below.

The MS of **1** provided a nominal mass of *m*/*z* 391 [M + H]^+^, initially suggesting a molecular formula typical for a diisooctyl phthalate contaminant (C_24_H_38_O_4_). However, an isotope ratio fitting calculation eliminated this possibility, and the molecular formula C_23_H_38_N_2_OS was derived (*m*/*z* 391.2778, calcd.; six degrees of unsaturation). A preliminary analysis of ^1^H- and ^13^C-NMR spectra of **1** in C_6_D_6_ showed resonances for two deshielded methyl groups, an amide *N*-methyl group in two equilibrating tautomeric configurations [δ_H_ 2.90 (s) and 2.35 ppm (s) (3H together, CH_3_-21)] and a methyl group attached to an sp^2^ carbon [δ_H_ 1.98 (3H, s, CH_3_-23)]. Additionally, eight sp^2^ carbons were observed for **1**, forming one mono-substituted vinylidene moiety, two di-substituted *trans* alkenes, and two carbonyl or imine functional groups. At this point, the remaining degree of unsaturation could be explained by either the presence of a ring structure or a carbon-nitrogen triple bond.

**Figure 4:**
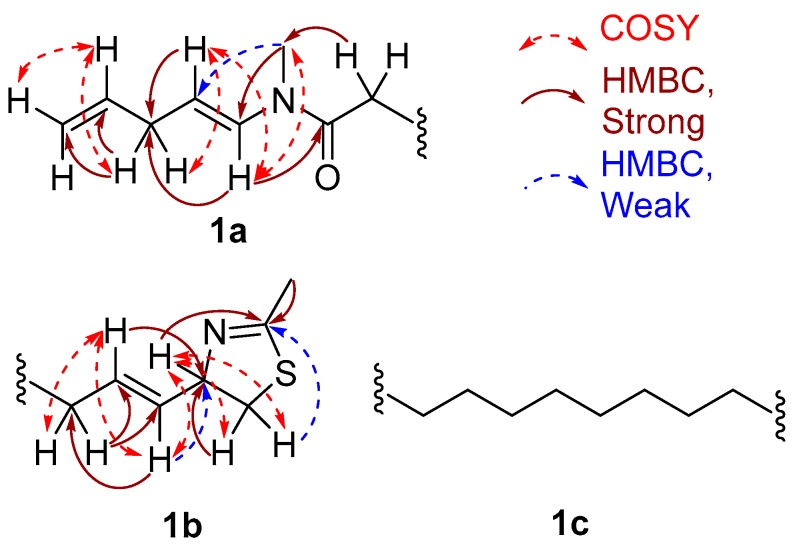
Selected COSY and HMBC correlations for **1a** and **1b**, two partial substructures of laucysteinamide A (**1**), plus the intervening substructure **1c**.

**Figure 5:**
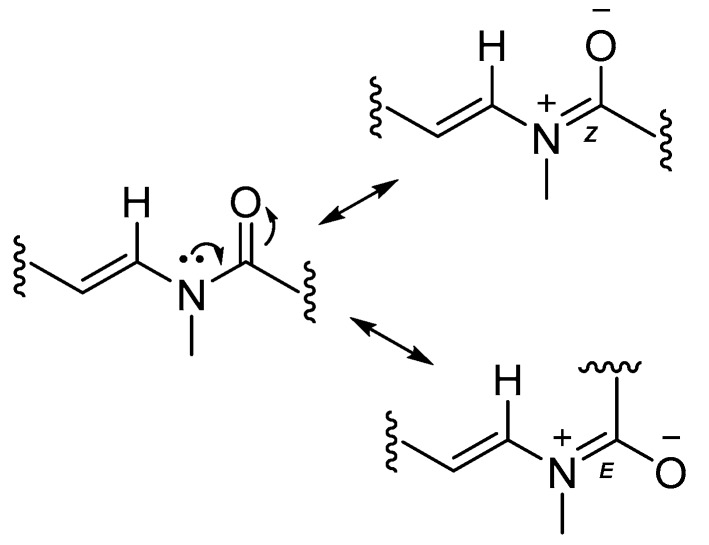
The imine-enamine tautomerism results in two sets of chemical shifts for atoms in this region of laucysteinamide A (**1**).

The substructures of an amide (**1a**) and a 2-methyl-4-thiazolinyl ethenyl moiety (**1b**) were constructed according to interpretation of 1D and 2D NMR data obtained from ^1^H-^1^H Correlation Spectroscopy (COSY) and ^1^H-^13^C Heteronuclear Single Quantum Coherence (HSQC), Heteronuclear 2 Bond Correlation (H2BC), and Heteronuclear Multiple Bond Correlation (HMBC) experiments (Figure 4). The HSQC data allowed for the assignment of the combined three-proton signals at δ_H_ 2.35 and 2.90 to the *cis*/*trans* isomers of the *N*-methyl amide carbon (δ_C_ 31.4 and 29.6, C-21), respectively. The HMBC correlations from δ_H_ 2.35 to the sp^2^ methine and carbonyl carbons, δ_C_ 129.0 (δ_H_ 7.80, d, *J* = 14.4 Hz, C-16) and 170.5 (C-15), respectively, were not observed in the ^1^H-^13^C H2BC spectrum, thus suggesting the occurrence of an *N*-methyl enamide moiety. For the configurational isomer with CH_3_-21 at δ_H_ 2.90, HMBC correlations were observed with analogous carbons at δ_C_ 130.1 (δ_H_ 6.42, d, *J* = 13.8 Hz) and 170.2. By COSY and *J*-coupling analysis, the H-16 methine resonances were located adjacent to another set of divided proton signals [δ_H_ 4.61 (dt, *J* = 13.8, 6.6 Hz) and 4.66 (dt, *J* = 14.4, 7.2 Hz) (1H together, H-17)], with corresponding carbon resonances at δ_C_ 107.2 and 106.9. The magnitude of the *J*-coupling between H-16 and H-17 suggested a *trans*-double bond configuration. A deshielded methylene group [δ_H_ 2.60 (t, *J* = 6.1 Hz) and 2.68 (t, *J* = 6.1 Hz) (2H together, H-18), δ_C_ 34.7] was adjacent to C-17 according to H2BC correlations. ^1^H-^1^H COSY data sequentially connected the protons of CH_2_-18, alkenyl CH-19 [(δ_H_ 5.78 (m) and 5.80 ppm (m) (1H together), δ_C_ 137.5 and 138.0] and the alkenyl terminus CH_2_-20 [δ_H_ 5.03 (m, 2H), 114.9 and 115.2]. Completing partial structure **1a** was a moderately deshielded methylene group at δ_H_ 2.09 (2H, t, *J* = 7.2 Hz), δ_C_ 33.6), located next to the carbonyl by HMBC and H2BC correlations. The two sets of ^1^H-NMR and ^13^C-NMR chemical shift data for each carbon and proton from C-14 to C-21 is explained by conformational anisotropy caused by *cis*/*trans* isomerism of the corresponding *N*-methyl quaternary imine (Figure 5), as described previously [6,9]. The *cis*/*trans* ratio between these isomers (0.44:1) was calculated in C_6_D_6_ at 20 °C using peak integrals from the ^1^H-NMR spectrum.

The remaining unassigned methyl signal from the ^1^H-NMR spectrum [δ_H_ 1.99 ppm (3H, s, CH_3_-23)] showed a strong HMBC correlation with an imine carbon [δ_C_ 164.7 ppm (C-22)]. A heteroatom-substituted methylene group [δ_H_ 3.01 (1H, dd, *J* = 10.8, 8.4 Hz, H-1a) and 2.79 (1H, dd, *J* = 10.8, 8.4 Hz, H-1b)] and a more deshielded methine moiety [δ_H_ 4.80 (1H, ddd, *J* = 6.6 Hz, CH-2), δ_C_ 79.2] also showed HMBC corrections with this imine carbon. By COSY and H2BC, the H_2_-1 protons were shown to be adjacent to the C-2 methine, and this could be extended to an alkenyl methine group [δ_H_ 5.53 (1H, dd, *J* = 15.6, 6.6 Hz, H-3)]. Proton H-3 showed COSY and H2BC correlations with another vinyl proton [δ_H_ 5.69 (1H, dd, *J* = 15.6, 7.8 Hz, H-4); δ_C_ 132.4 (C-4)]; the large *J*-value between H-3 and H-4 indicated a *trans*-relationship. This latter CH group was adjacent to an allylic methylene group (C-5) as revealed by multiple COSY and HMBC correlations (Figure 4, Table 1). The positioning of a sulfur between C-23 and C-1 and nitrogen atom between C-23 and C-2 was supported by comparison with the ^1^H- and ^13^C-NMR data in benzene-*d*_6_ for the 2-alkyl-4-ethenyl-thiazoline moiety present in both curacin D [5] and curacin A [10]. Altogether, these data suggested the presence of a methylene-substituted 2-methyl-4-ethenyl-thiazoline subunit in laucysteinamide A (**1**) (Figure 1).

The two partial structures, **1a** and **1b**, were connected by a saturated linear alkyl chain (**1c**, C-6 to C-13) to form the final planar structure of compound **1**. The insertion of eight methylene groups between C-5 and C-14 satisfied the molecular formula, and was supported by COSY and HMBC correlations between the terminal atoms of partial structures **1a** and **1b** and the shielded methylene envelope of resonances of partial structure **1c** (Figure 4). Consequently, the planar structure of laucysteinamide (**1**) was shown to be an alternately condensed form of a monomer of the symmetrical dimeric metabolite somocysteinamide A (**3**) [6].

### 2.1. Stereochemistry

**Figure 6:**
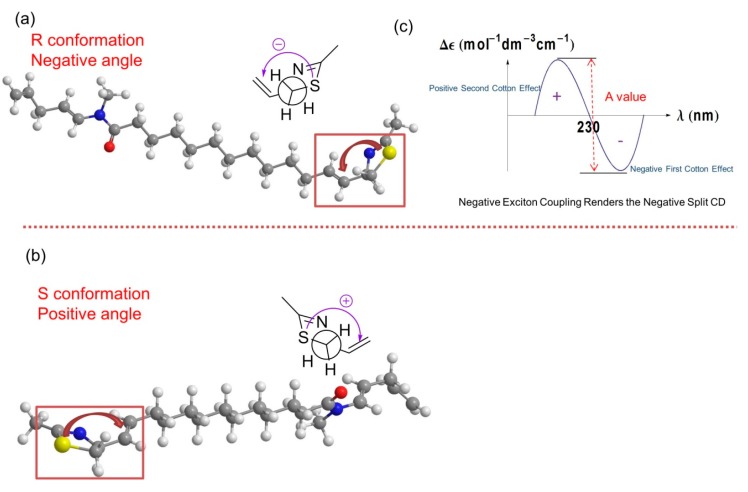
Molecular Modeling and exciton coupling circular dichroism (ECCD) prediction. (**a**) The stick-ball model of the *R* configuration of compound **1** and its Newman projection of the relevant thiazoline moiety. The angle between the two chromophores in the model is negative (counterclockwise). Atom definitions: red, oxygen; yellow, sulfur; blue, nitrogen; gray, carbon; white, hydrogen; (**b**) The stick-ball model of the *S* configuration of compound **1** and its Newman projection of the relevant thiazoline moiety. The angle between the two chromophores in the model is positive (clockwise). Atom definitions as in panel a; (**c**) The *R* conformation of compound **1** with negative exciton coupling renders the negative split ECCD curve as indicated by the experimental result shown in Figure S11.

The absolute configuration of laucysteinamide A was determined by comparison of energy-minimized molecular models of **1** with observed exciton coupling circular dichroism (ECCD) data. Computational molecular models of enantiomeric forms of **1** were subjected to energy minimization with MOPAC software (ChemBioDraw Ultra 13.0, PerkinElmer Inc., Waltham, MA, USA) [11], the results of which are shown in Figure 6. The through-space coupling of nearby interacting chromophores gives rise to diagnostic angle-dependent exciton coupling in the circular dichroism spectrum [12]. In the case of laucysteinamide A, a thiazoline chromophore is present in the vicinity of the C-3/C-4 alkenyl moiety. The coupling of these chromophores gives rise to a corresponding split Cotton effect, as shown in Figure 6. The ECCD spectrum of compound **1** (Figure S11) showed a negative local maximum at 223 nm, corresponding to the thiazoline chromophore. The maximum expected from the C-3/C-4 alkene would be around 190 nm, but was not observed in the spectrum due to solvent absorptions. However, the CD spectrum clearly showed a negative first Cotton effect, and thus, the absolute configuration of compound **1** is confidently assigned as 2*R*. Compound **2**, which is an analogue of compound **1**, was assigned previously with a 2*R*,2′*R* absolute configuration, and it showed a similar optical rotation ${\left[\alpha \right]}_{D}^{22}$ = +13.5° (*c* = 0.75, CHCl_3_) to compound **1**, thus providing additional support for the absolute configuration of compound **1** as 2*R* [6].

### 2.2. Bioactivity

Although laucysteinamide A (**1**) is structurally analogous to somocystinamide A (**2**), which was previously shown to be a potent inhibitor of angiogenesis and cancer cell proliferation (IC_50_ = 3 nM against Jurkat leukemia cells [1]), **1** was only mildly cytotoxic to H-460 human non-small cell lung cancer cells (IC_50_ = 11 μM), compared with doxorubicin (EC_50_ = 0.032 μM). Thus, the cytotoxic activity of this fraction (fraction D) was almost entirely due to the antitubulin properties of curacin D (**3**) [5]. The brine shrimp toxicity data of all VLC fractions and crude extract are shown in Table S1 in the supplementary information; unfortunately, compound **1** decomposed before it could be evaluated for this biological activity.

### 2.3. Biosynthetic Considerations

Based on its essentially linear structure, we propose that laucysteinamide A is assembled by a hybrid PKS/NRPS pathway as it possesses sections logically deriving from amino acid residues (cysteine and *N*-methylglycine) and nine acetate units (Figure S12). The assembly of **1** could be initiated by loading an acetate unit followed by an NRPS-mediated condensation with cysteine, heterocyclization and dehydration to form the thiazoline ring, followed by six cycles of polyketide extension. While the first ketide extension is only partially reduced, the ensuing five are fully reduced. Next, another NRPS module is envisaged to incorporate a glycine residue, followed by an *S*-adenosyl methionine assisted methylation of the glycine *N*-atom (Figure S12). After two more cycles of acetate extension, the chain termination (Figure S12) likely concludes with a sulfotransferase-aided sulfonate esterification of the β-hydroxy group, thioesterase hydrolysis and then coincident decarboxylation and sulfate elimination. This termination sequence is identical to that found in the previously described curacin A pathway [13].

## 3. Experimental Section

### 3.1. General Experimental Procedures

Optical rotations were recorded with a JASCO P-2000 polarimeter; ECCD spectra were measured in CH_2_Cl_2_ using a JASCO J-810 spectropolarimeter (Jasco, Easton, MD, USA). UV spectra were recorded with a Beckman Coulter DU800 spectrophotometer (Indianapolis, IN, USA); IR spectra were collected on a Bruker ALPHA-P FTIR spectrometer with a diamond ATR (Bruker Optics, Billerica, MA, USA). ^1^H NMR and 2D NMR spectra of laucysteinamide A (**1**) were measured on the Bruker 600 MHz NMR spectrometer with a 5 mm inverse detection triple resonance (^1^H-^13^C/^15^N/^2^D) cryoprobe (Bruker Biospin, Billerica, MA, USA). The remaining ^1^H- and ^13^C-NMR spectra were measured on a JEOL ECA 500 MHz spectrometer (JEOL, Akishima, Tokyo, Japan) or Varian XSens 2 channel (^1^H/^13^C) NMR cryoprobe optimized for direct observation of ^13^C-NMR, with samples dissolved in CDCl_3_ or C_6_D_6_ (Varian, Palo Alto, CA, USA). Low-resolution MS spectra were recorded on a Thermo Finnigan LCQ, operating in positive ion ESI mode, coupled to a Thermo Finnigan Surveyor Plus liquid chromatography system (Thermo Scientific, Waltham, MA, USA). HRMS data were obtained with an Agilent 6230 TOF-MS (Agilent, Santa Clara, CA, USA) under positive ion ESI-TOF-MS conditions and provided by the University of California, San Diego (UCSD) Small Molecule MS Facility. Semipreparative HPLC was carried out using a Waters 515 pump system with a Waters 996 PDA detector (Waters Corporation, Milford, MA, USA).

### 3.2. Sample Material

A marine cyanobacterial sample, assigned the code SPL-3FEB13-2, was collected in February 2013 from shallow water in Lau Lau Bay, Saipan (GPS coordinates: 15°09′35.5′′ N and 145°45′25.5′′ E). The taxonomy of the collected sample was determined to be cf. *Caldora penicillata* by microscopic characterization. The 2.5–15 cm long cyanobacterial filaments possessed a mucilagenous base and grew mainly from the tops of *Turbinaria* sp. The tan/purple colored sample was preserved in approximately 750 mL of isopropanol in sea water at −20 °C prior to laboratory extraction. A voucher specimen is preserved and available from the Gerwick Voucher Collection, Scripps Institution of Oceanography, University of California San Diego.

### 3.3. Extraction and Isolation

The sample biomass was defrosted and then extracted with DCM/MeOH (2:1), eight times, to yield 6.73 g of dark green crude extract. The extracted cyanobacterial biomass was 71.3 g in dry weight. This crude extract was subjected to a stepped-gradient fractionation (hexanes/EtOAc and EtOAc/MeOH) by vacuum liquid chromatography (VLC) over normal phase silica gel to give ten fractions (A–J). Fraction C (eluted with 20% EtOAc/hexanes) was again separated by normal phase chromatography on silica gel with a stepwise gradient of hexanes/EtOAc to give six sub-fractions (Ca-Cf). Fraction Cb was further purified by chromatography on a normal-phase Luna HPLC column (100 Å, 5 μm, 250 × 1000 mm, isocratic solvent system comprised of 10% EtOAc/hexanes over 45 min; flow rate 3 mL/min; PDA detection) to give 2.7 mg of compound **3** (*t*_R_ = 13.2 min), which had a pale yellowish color. The ^1^H- and ^13^C-NMR spectra of **3** in C_6_D_6_ matched literature reported values for curacin D [5]. The optical rotation value of **3**, ${\left[\alpha \right]}_{D}^{25}$ = +33.3° (*c* = 0.14, CHCl_3_), matched the reported value ${\left[\alpha \right]}_{D}^{25}$ = +33° (*c* = 0.14, CHCl_3_) [5].

The ^1^H-NMR spectrum of fraction D in CDCl_3_ showed peaks with coupling patterns in the 4.0 ppm to 7.5 ppm region, and was selected for further investigation. The major peak in this fraction by LC-MS/MS analysis showed an [M + H]^+^ at *m*/*z* 391.11; by MarinLit searching and Molecular Network analysis, this compound did not correlate with any known compound. Fraction D was further separated with HPLC as above on a normal-phase Luna column (100 Å, 5 μm, 250 × 1000 mm, solvent system of a linear gradient starting with 100% 1:3 EtOAc/hexanes for 25 min before being ramped to 100% 1:1 EtOAc/hexanes in 10 min followed by maintenance in 100% 1:1 EtOAc/hexanes for another 5 min; flow rate 3 mL/min; PDA detection at 254 nm), giving rise to 3.6 mg of compound **1** (*t*_R_ = 34.5 min).

### 3.4. Molecular Networking

The fractions and crude extract were each diluted to 1 mg/mL in MeOH for LC-MS with automated dependent MS/MS scanning. The following gradient was used: initiated in 50% CH_3_CN in MilliQ H_2_O with 0.1% formic acid (*v*/*v*) for 4 min, then CH_3_CN increased to 99% in a linear gradient over 12 min and then maintained at this percentage for 5 min. Finally, the CH_3_CN was reduced to 50% and stabilized for 3 min. MS data from two scan events were acquired: (1) scan positive MS, window from *m*/*z* 190–2000; (2) scan MS/MS in data-dependent mode for the most intense ions from the first scan. The raw Thermo XCalibur data files were processed with MSConvert to produce.mxz files, and these were submitted for molecular networking using the GNPS platform [14]. The resulting molecular networks were graphically represented using Cytoscape [15].

### 3.5. Biological Testing

In vitro cytotoxicity studies were performed using H-460 human non-small cell lung cancer cells as previously described [16]. Briefly, H-460 cells were added to 96-well plates at 3.33 × 10^4^ cells/mL of Roswell Park Memorial Institute (RPMI) 1640 medium with fetal bovine serum (FBS) and 1% penicillin/streptomycin. The cells were incubated overnight (37 °C, 5% CO_2_) in a volume of 180 μL per well to allow recovery before treatment with test compounds. Compounds were dissolved in dimethyl sulfoxide (DMSO) to a stock concentration of 1 mg/mL. Working solutions were made through serial dilution in RPMI 1640 medium without FBS, with 20 μL added to each well producing final compound concentrations of 10, 3, 1, 0.3, 0.1, 0.03, 0.01, 0.003, and 0.001 μg/mL. An equal volume of RPMI 1640 without FBS was added to wells designated as negative controls for each plate. Plates were incubated for approximately 48 h before MTT staining. Plates were read at 570 and 630 nm using a Thermo Electron Multiskan Ascent plate reader (Thermo Scientific, Waltham, MA, USA).

Brine shrimp toxicity studies were performed as previously described [17]. Briefly, brine shrimp eggs were hatched for 24 h in brine solution. Each well of the bioassay plate was prefilled with 2 mL brine solution, 200 μL brine shrimp culture mixture (around 15 brine shrimp in brine solution), and 300 μL brine solution, sequentially. Test fractions and compounds were dissolved and added to wells in 10 μL DMSO to afford a final concentration of 3 μg/mL and 30 μg/mL in each well, in duplicate per concentration. A sample of 10 μL DMSO was added to separate wells as a negative control. After 24 h, the number of dead non-moving brine shrimp was counted with the aid of a dissecting microscope. Acetone (1.2 mL) was added to each well to sacrifice the shrimp and the total number of dead shrimp was counted. The difference in the two counts represents the number of live shrimp at the end of the test period.

*Laucysteinamide A* (**1**): pale yellowish oil. ${\left[\alpha \right]}_{D}^{26}$ = +17.07° (*c* = 0.86, CHCl_3_); UV λ_max_ (CH_2_Cl_2_) 223 nm; IR (neat) λ_max_ 2927, 2844, 1662, 1634, 1464, 1394, 1338, 1161, 1087, 912 cm^−1^; ^1^H-, ^13^C-, and 2D-NMR see Table 1; ESIMS *m*/*z* 391 [M + H]^+^; HRESITOFMS *m*/*z* 391.2777 [M + H]^+^ (calcd. for C_23_H_39_N_2_OS, 391.2778).

*Curacin D* (**3**): pale yellow oil. ${\left[\alpha \right]}_{D}^{25}$ = +33.3° (*c* = 0.14, CHCl_3_), Lit. ${\left[\alpha \right]}_{D}^{25}$ = +33° (*c* = 0.14, CHCl_3_) [5]; UV λ_max_ (hexanes) 224 (ε 9000) nm ^1^H-NMR (C_6_D_6_, 500 MHz) δ 6.1 (1H, m, H-9), 6.0 (1H, m, H-8), 5.79 (1H, ddt, *J* = 16.2, 11.0, 7.2 Hz, H-15), 5.64 (1H, dd, *J* = 10.5, 10.4 Hz, H-3), 5.55 (1H, dt, *J* = 14.5, 7.1 Hz, H-10), 5.49 (1H, bdt, *J* = 14.5, 7.3 Hz, H-7), 5.38 (1H, m, H-4), 5.05 (2H, m, H-16), 5.03 (1H, m, H-2), 3.12 (3H, s, -OMe), 3.05 (1H, m, H-13), 3.03 (1H, dd, *J* = 10.3, 8.3 Hz, H-1b), 2.74 (1H, dd, *J* = 10.3, 10.3 Hz, H-1a), 2.2 (4H, m, H-11,14), 2.1 (2H, m, H-5), 2.0 (2H, m, H-6), 1.67 (1H, td, *J* = 8.3, 5.5 Hz, H-18), 1.58 (1H, m, H-12a), 1.54 (1H, m, H-12b), 1.17 (3H, d, *J* = 6.3 Hz, H-21), 1.15 (1H, m, H-19b), 0.95 (1H, m, H-20), 0.67 (1H, ddd, *J* = 8.1, 8.1, 4.3 Hz, H-19a); ^13^C-NMR (C_6_D_6_, 100 MHz) δ 168.44 (C17), 135.09 (C15), 132.29 (C10), 131.39 (C9), 131.12 (C3), 131.05 (C7), 130.87 (C8), 130.57 (C4), 116.54 (C16), 79.56 (C13), 74.1 (C2), 56.05 (OMe), 39.69 (C1), 37.91 (C14), 33.39 (C12), 32.51 (C6), 28.51 (C11), 27.67 (C5), 19.86 (C18), 15.71 (C20), 13.96 (C19), 12.06 (C21); LC LRMS [M + H]^+^ *m*/*z* 360.114.

## 4. Conclusions

Marine cyanobacteria, such as *Caldora penicillata*, are a continuing source of new and bioactive molecules of interest for natural product drug discovery research. A novel thiazoline containing alkaloid, laucysteinamide (**1**), along with known compounds, curacin D (**3**), were isolated from the marine cyanobacterium, cf. *Caldora penicillata*, collected from island of Saipan. Laucysteinamide A (**1**) was determined to be mildly cytotoxic to H-460 human non-small lung cancer cells, and was isolated together with the known potent bioactive molecule, curacin D (**3**), which was initially identified by LC-MS/MS molecular networking dereplication efforts. Laucysteinamide A (**1**) belongs to a unique cyanobacterial metabolite class of mixed PKS/NRPS biosynthetic origin, analogous to the previously reported somocystinamide A (**2**). We propose that the acetated l-cysteine is extended with six more malonyl CoA-derived acetate units. This intermediate is then further extended with an *N*-methyl glycine unit, followed by two additional acetates. The proposed biosynthesis of **1** is terminated by a decarboxylation very similar to that described for curacin A [14].

## Figures and Tables

**Table 1:** NMR Spectroscopic Data for Laucysteinamide A (**1**) in Benzene-*d*_6_.

Position	δ_H_ Mult. (*J* in Hz)	δ_C_, Mult. (*J* in Hz)	COSY	H2BC	HMBC
1	3.01, dd (10.8, 8.4)	40.3	2.79, 4.80	79.2	32.7, 79.2, 164.7
	2.79, dd (10.8, 8.4)		3.01, 4.80	79.2	164.7
2	4.8, ddd (6.6)	79.2	2.79, 3.01, 1.97, 5.53	40.3	164.7, 130.1, 132.4
3	5.53, dd (15.6, 6.6)	130.1	1.97, 4.80	79.2, 132.4	32.7, 132.4
4	5.69, dt (15.6, 7.8)	132.4	1.97	32.7, 130.1	29.9, 32.7, 130.1
5	1.97, m	32.7	4.80, 5.53/5.69	132.4	40.3
6	1.25, m	29.9			30.0
7 to 12	1.25–1.4, m	30.0			
13	1.69, m	25.3	1.28, 1.93, 2.09	33.6	30.0
14	2.09, t (7.2)	33.6	1.69	25.3	30.0
	1.93, t (7.8)	34.7	1.69	25.3	170.2
15		170.5			
		170.2			
16	6.42, d (13.8)	130.1	4.61	107.2	34.7, 29.6, 170.5
	7.80, d (14.4)	129.0	4.66	106.9	31.4, 34.7
17	4.61, dt (13.8, 6.6)	107.2	2.60, 6.42	129.0	34.7/34.8, 34.7, 129.0, 137.5
	4.66, dt (14.4, 7.2)	106.9	2.68, 7.80	137.5	34.7/34.8
18	2.60, t (6.1)	34.7	4.61, 5.78	107.2/106.9	114.9/115.2, 107.2, 130.1, 137.5
	2.68, t (6.1)	34.8	4.66, 5.80		114.9/115.2, 106.9, 138.0
19	5.78, m	137.5	2.60, 5.03	114.9	130.1
	5.80, m	138.0	2.68, 5.03	115.2	129.0
20	5.03, m	114.9	2.60/2.68	137.5/138.0	34.7/34.8
	5.03, m	115.2	2.60/2.68	137.5/138.0	34.7/34.8
21	2.90, s	29.6	6.48		130.1, 107.2/106.9, 170.5
	2.35, s	31.4			129.0, 170.5
22		164.7			79.2
23	1.98, s	20.3			164.7

## Supplementary Materials

The following are available online at www.mdpi.com/1660-3397/15/4/121/s1, Figure S1: (a) Fragmentation analysis of curacin D (**3**). (b) MS of curacin D (**3**) (positive ion mode). (c) MS/MS (positive ion mode) spectra of curacin D (**3**), Figure S2: ^1^H NMR spectra of curacin D (**3**) in C_6_D_6_, Figure S3: HPLC chromatogram of laucysteinamide A (**1**), Figure S4: HRESITOFMS results of laucysteinamide A (**1**), Figure S5: ^1^H NMR spectra of laucysteinamide A (**1**) in C_6_D_6_, Figure S6: ^13^C NMR spectra of laucysteinamide A (**1**) in C_6_D_6_, Figure S7: COSY spectra of laucysteinamide A (**1**) in C_6_D_6_, Figure S8: ^1^H-^13^C HSQC spectra of laucysteinamide A (**1**) in C_6_D_6_, Figure S9: H2BC spectra of laucysteinamide A (**1**) in C_6_D_6_, Figure S10: HMBC spectra of laucysteinamide A (**1**) in C_6_D_6_, Figure S11: ECCD Spectrum of laucysteinamide A (**1**), Figure S12: Biosynthetic scheme proposed for laucysteinamide A, Figure S13: (a) Low resolution MS (positive ion mode) of laucysteinamide A (**1**). (c) MS/MS (positive ion mode) spectra of 1, Table S1: Brine Shrimp Assay Results of the 10 Fractions (A–J) and Crude Extract of the Sample.
