# Supplementary material for: Laucysteinamide A, a Hybrid PKS/NRPS Metabolite from a Saipan Cyanobacterium, cf. Caldora penicillata
Source: Mar Drugs. 2017 Apr 14;15(4):121. doi: 10.3390/md15040121 (PMC5408267; doi:10.3390/md15040121)
Supplement: Supplementary file 1 [file marinedrugs-15-00121-s001.pdf]

Supplementary Materials: Laucysteinamide A, a Hybrid PKS/NRPS Metabolite from a Saipan Cyanobacterium, cf. *Caldora penicillata* (Supplementary Materials)

Chen Zhang [1], C. Benjamin Naman [2], Niclas Engene [3] and William H. Gerwick [2,4*]

Table S1. Brine Shrimp Assay Results of the 10 Fractions (A-J) and Crude Extract of the Sample

Conc.	Tray #		A	B	C	D	E	F	G	H	I	J	Crude
3 μg/mL	1	death rate	-0.24	0.08	1	0.18	0	0	0.18	0	0	0	0
3 μg/mL	2	death rate	-0.1	0	1	0	0.14	0.06	0.11	0	0	0	0.06
		average	-0.17	0.04	1	0.09	0.07	0.03	0.15	0	0	0	0.03
30 μg/mL	3	death rate	0.25	1	0.95	1	1	0.89	0.71	0.86	0	0.14	1
30 μg/mL	4	death rate	0.29	0.83	1	1	1	1	1	0.79	0.09	0.12	1
		average	0.27	0.92	0.98	1	1	0.94	0.86	0.82	0.05	0.13	1

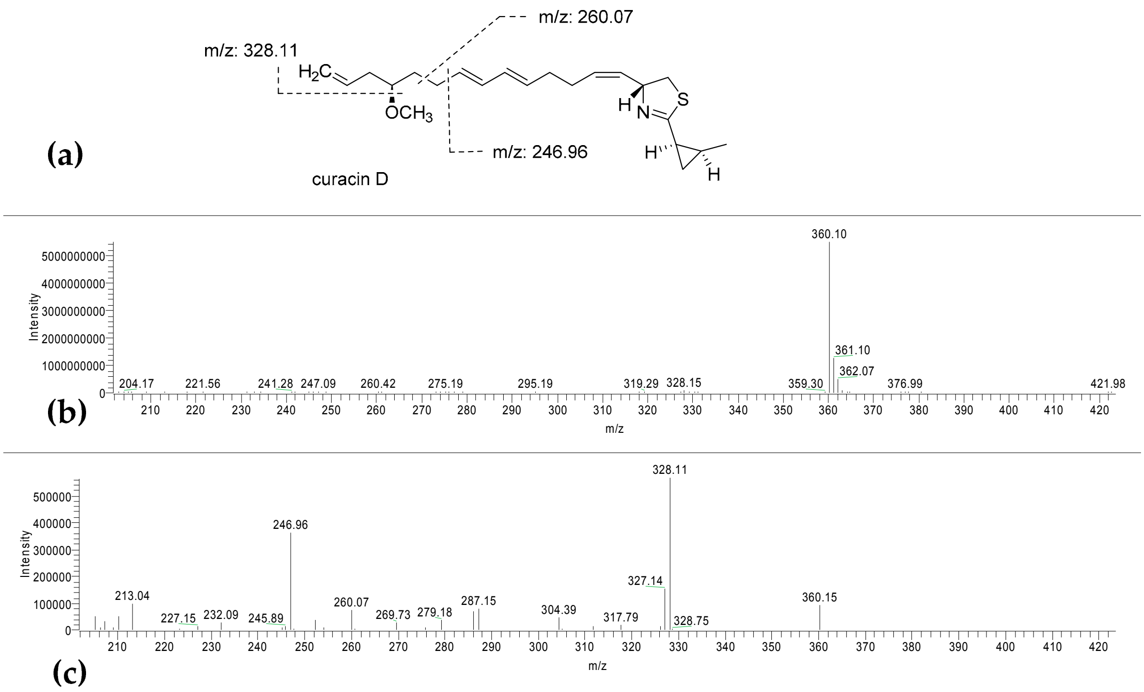

Figure S1. (**a**) Fragmentation analysis of curacin D (**3**). (**b**) MS of curacin D (**3**) (positive ion mode). (**c**) MS/MS (positive ion mode) spectra of curacin D (**3**).

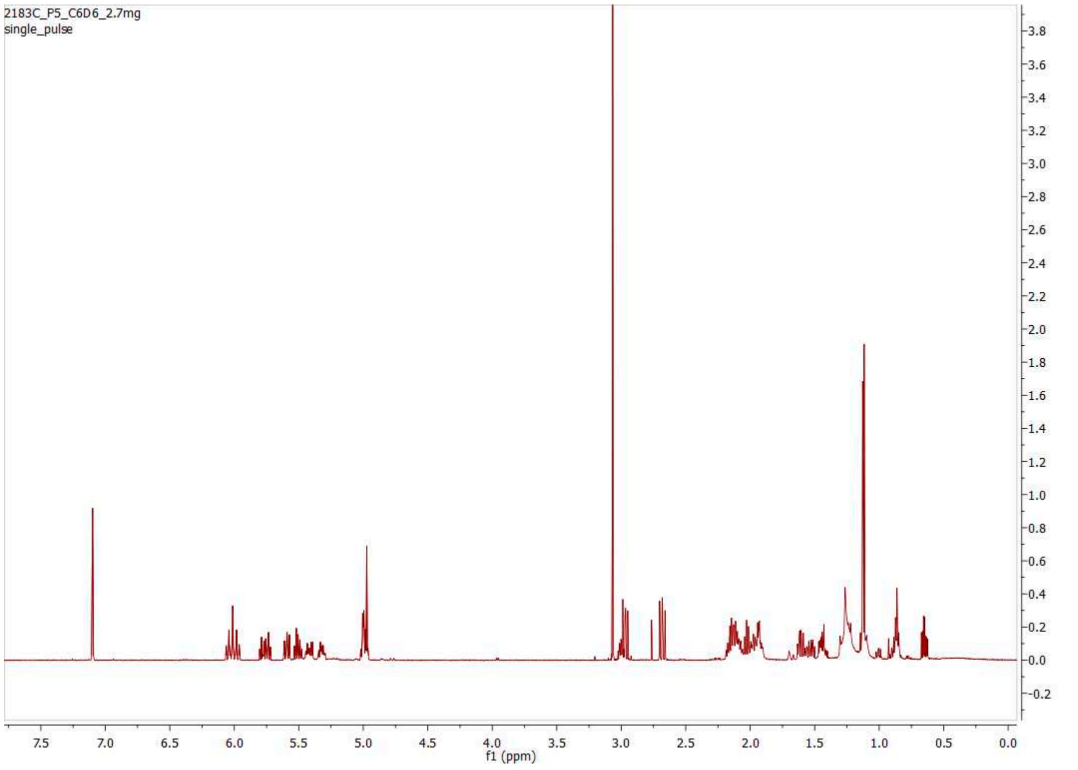

Figure S2. ^{1}H NMR spectra of curacin D (**3**) in C_6D_6.

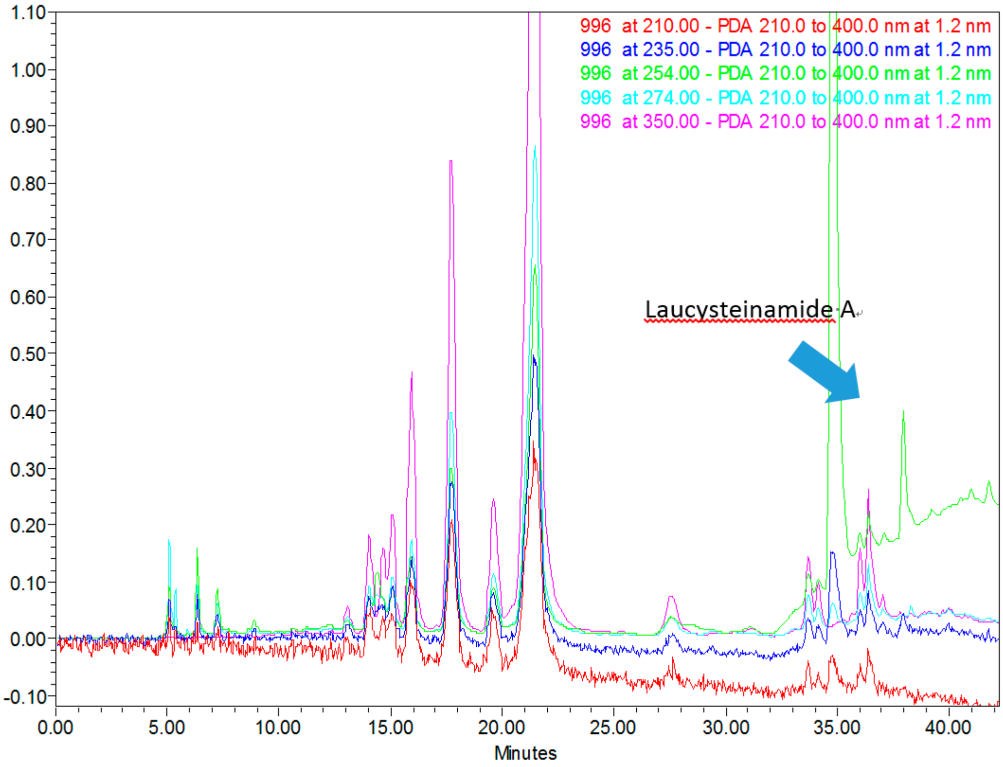

Figure S3. HPLC chromatogram of laucysteinamide A (**1**).

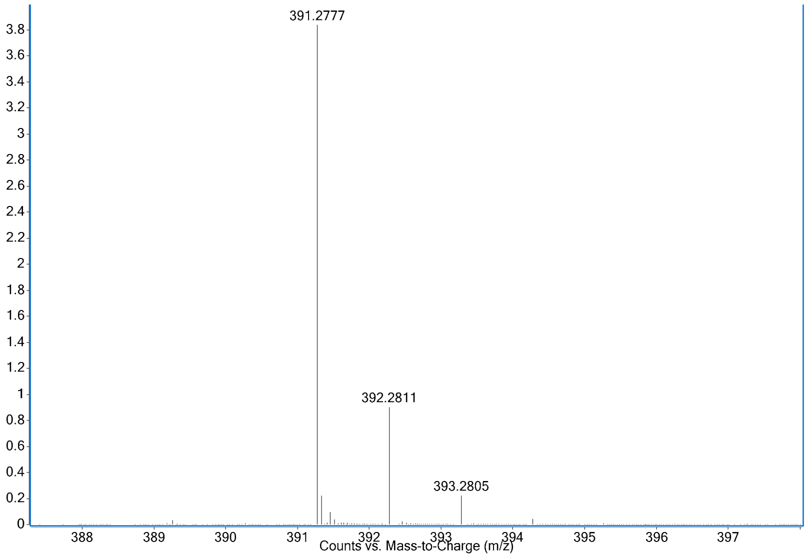

Figure S4. HRESITOFMS results of laucysteinamide A (**1**).

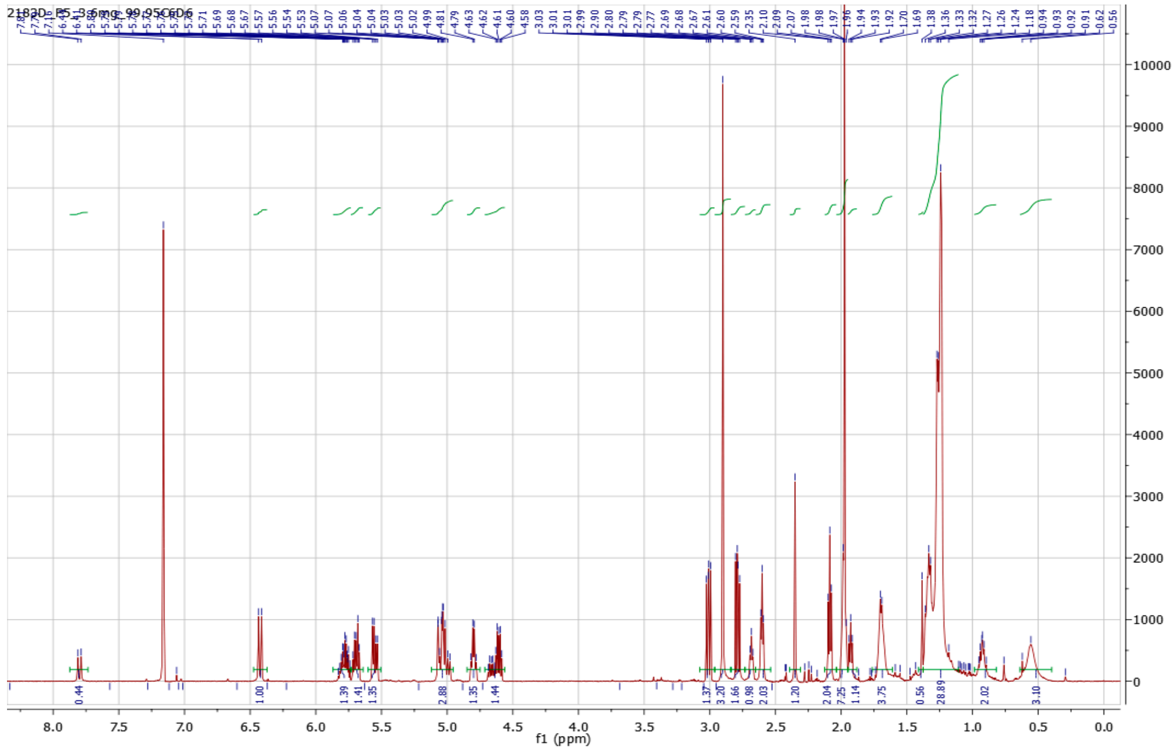

Figure S5. ^{1}H NMR spectra of laucysteinamide A (**1**) in C_6D_6.

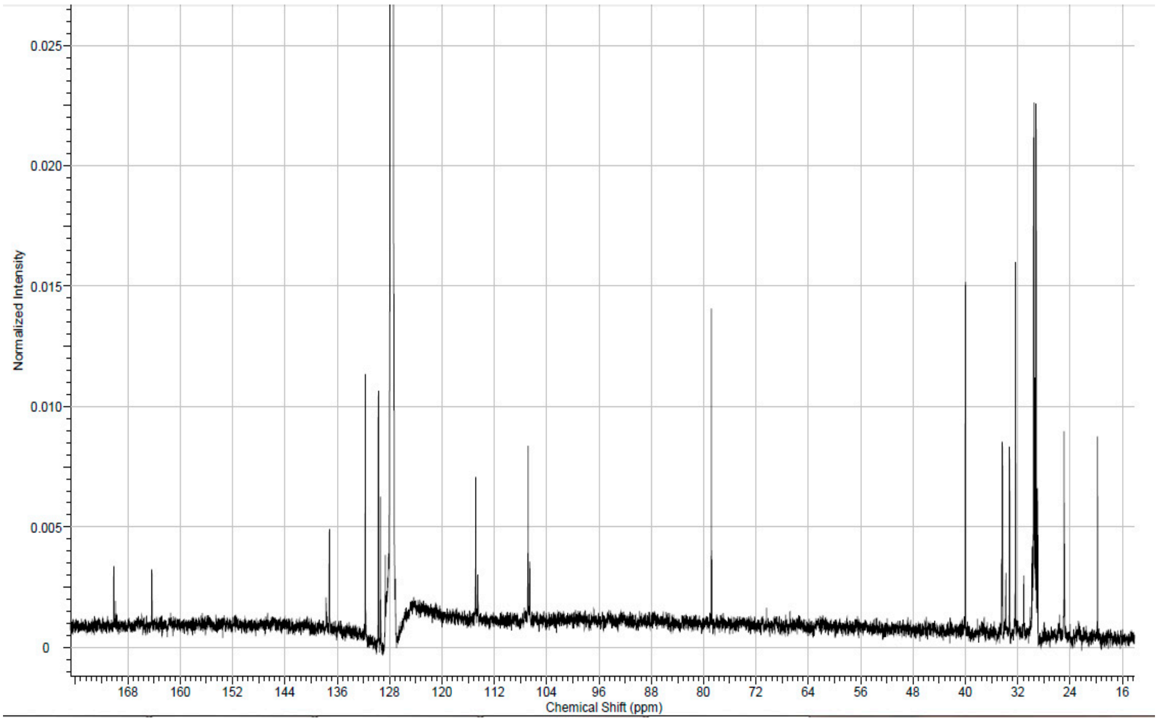

Figure S6. ^{13}C NMR spectra of laucysteinamide A (**1**) in C_6D_6.

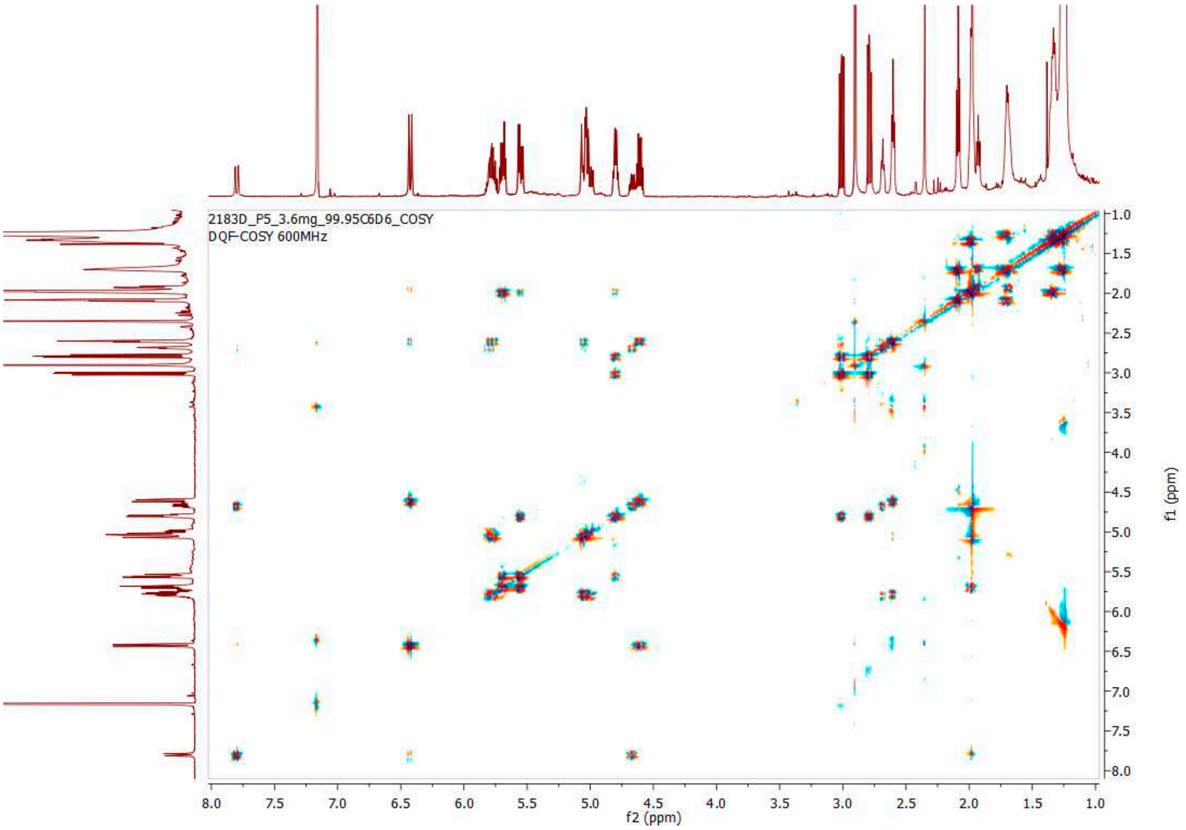

Figure S7. COSY spectra of laucysteinamide A (**1**) in C_6D_6.

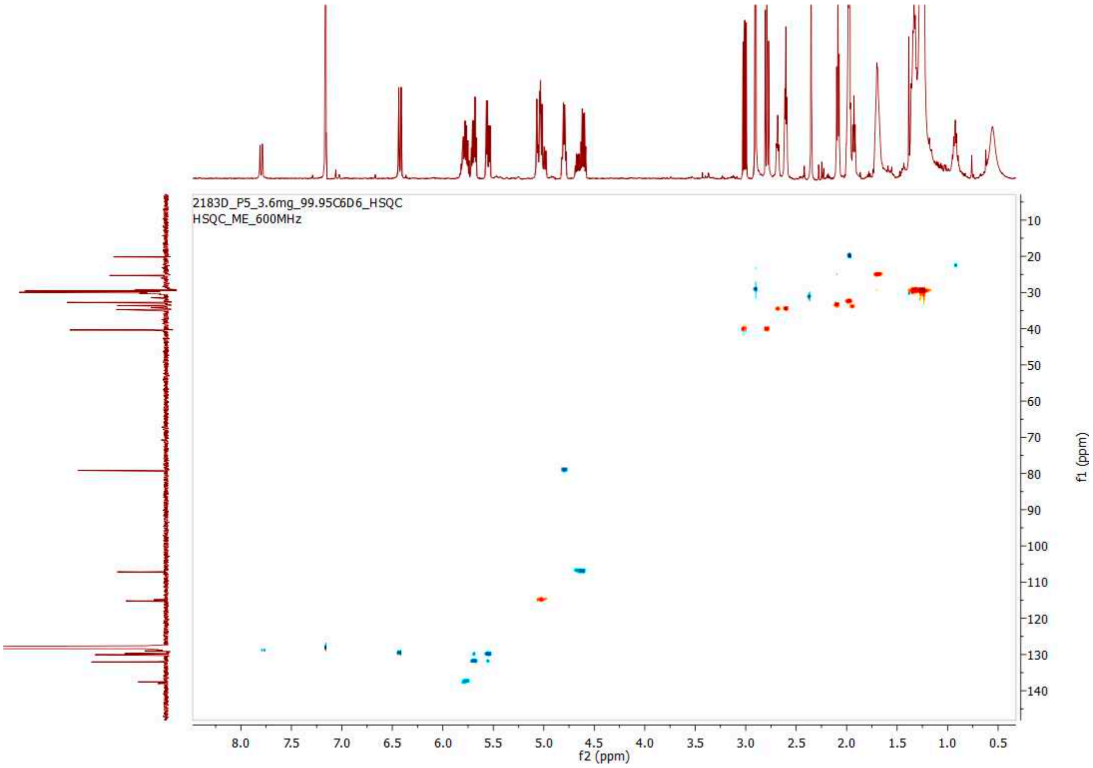

Figure S8. ^1H-^{13}C HSQC spectra of laucysteinamide A (**1**) in C_6D_6.

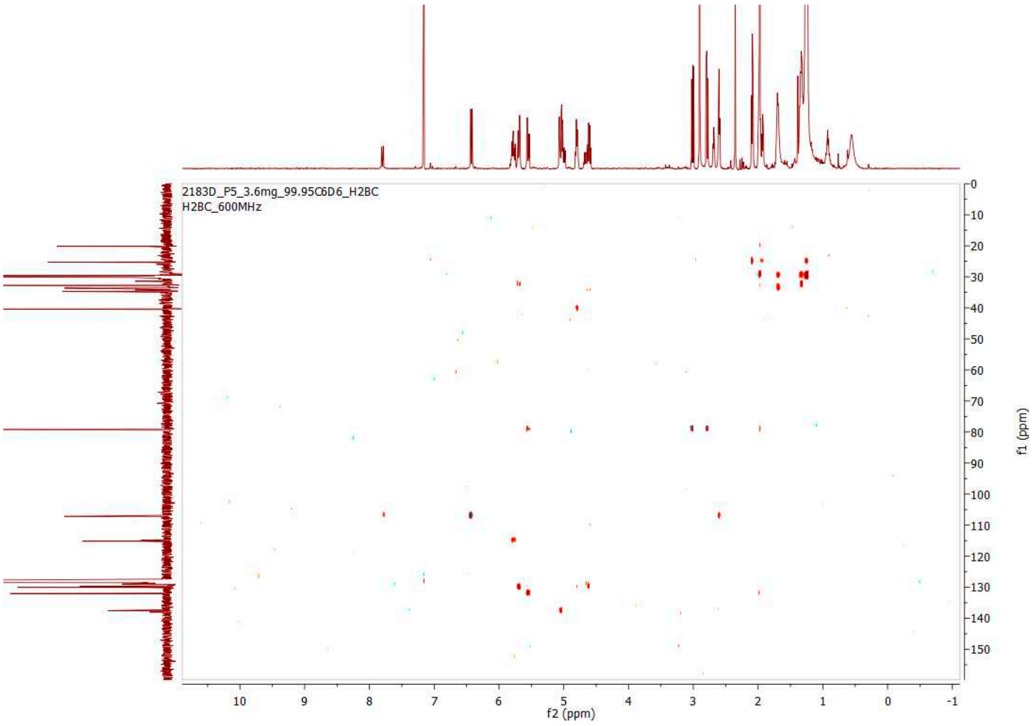

Figure S9. H2BC spectra of laucysteinamide A (**1**) in C_6D_6.

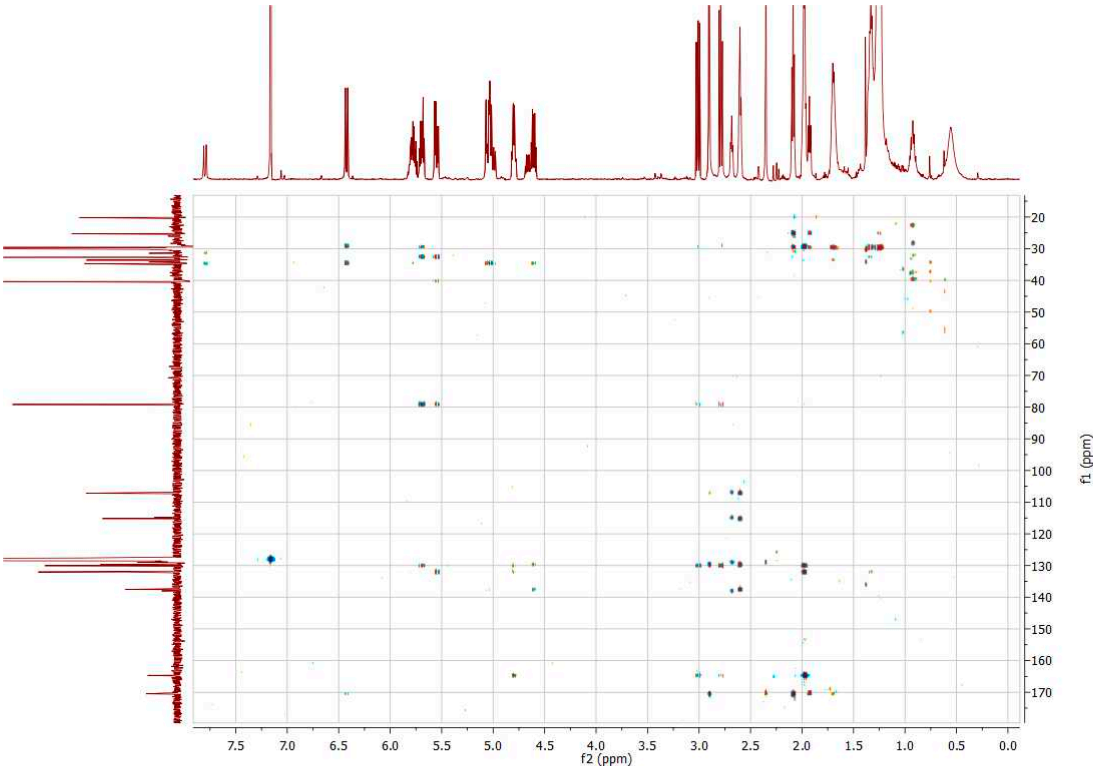

Figure S10. HMBC spectra of laucysteinamide A (**1**) in C_6D_6.

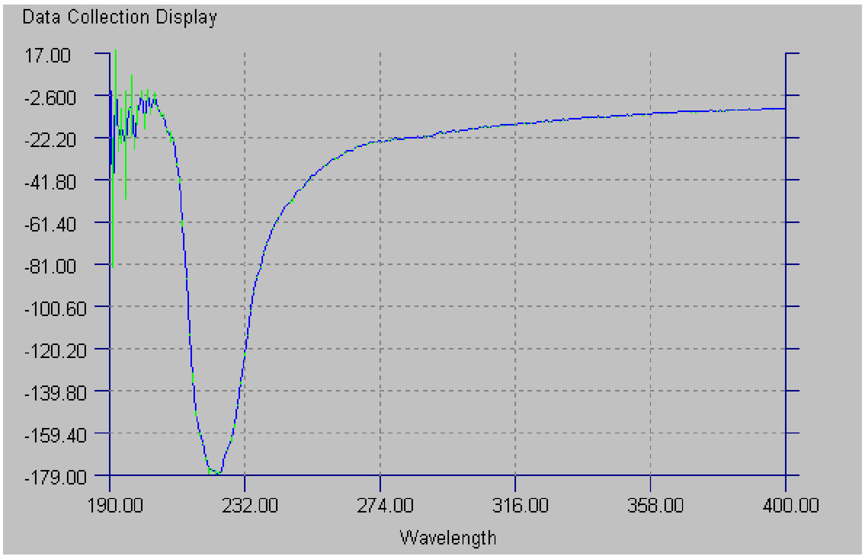

Figure S11. ECCD Spectrum of laucysteinamide A (**1**). The compound was dissolved in dichloromethane for the experiment. The region above 200 nm is obscured by solvent absorptions.

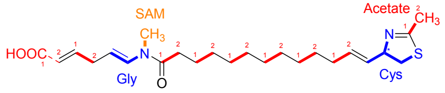
SAM
Acetate
HOOC
CH3
N
O
Gly
Cys
S

(a)

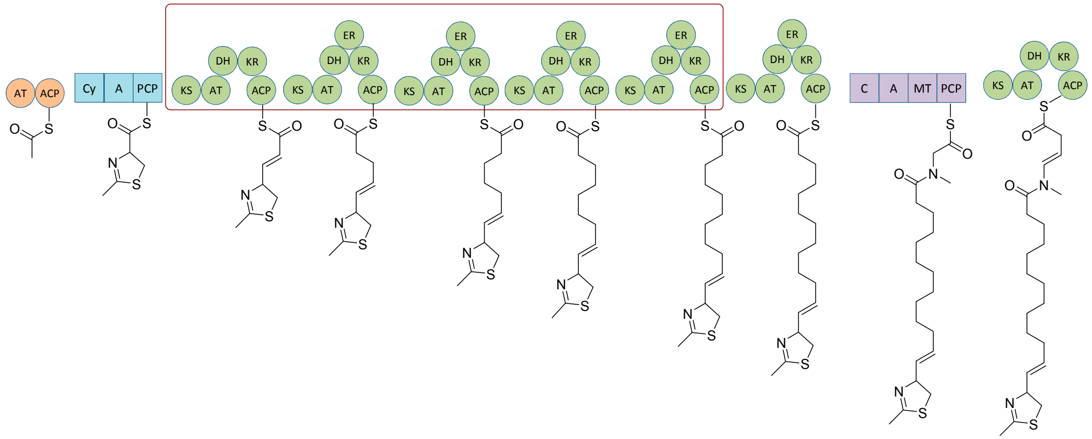
AT
ACP
Cy
A
PCP
KS
DH
KR
ER
C
MT

(b)

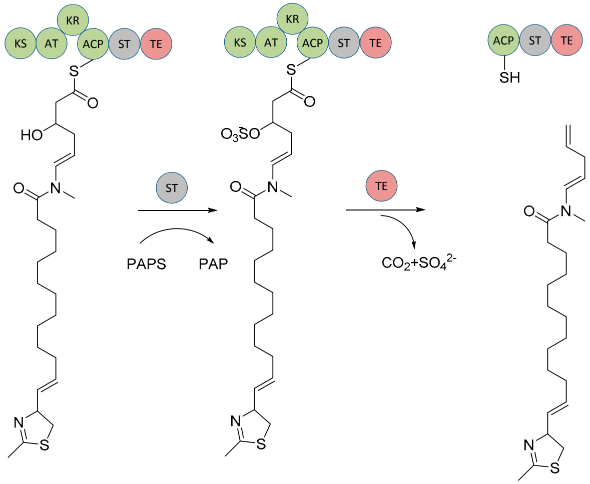
KS
AT
KR
ACP
ST
TE
HO
O3SO
PAPS
PAP
CO2+SO4 2-
SH

Figure S12. Biosynthetic scheme proposed for laucysteinamide A. (**a**) A hypothesized hybrid PKS/NRPS pathway of biosynthetic precursors. (**b**) The hybrid PKS/NRPS pathway prediction before chain termination with enzymatic domain. The last three steps show the predicted chain termination mechanism in laucysteinamide A (**1**) biosynthesis. This proposed biosynthetic pathway is based on that described for curacin A biosynthesis process[1,2]. Abbreviations: ACP, acyl carrier protein; KS, β-ketoacyl-ACP synthase; KR, β-ketoacyl-ACP reductase; AT, acyl transferase; DH, β-hydroxy-acyl-ACP dehydratase; ER, enoyl reductase; MT, N-methyl transferase; PCP, peptidyl carrier protein; Cy, condensaton/cyclization domain; A, adenylation domain; ST, sulfotransferase; PAPS, adenosine 3-phosphate 5-phosphosulfate; PAP, adenosine 3-phosphate 5-phosphate; TE, thioesterase.

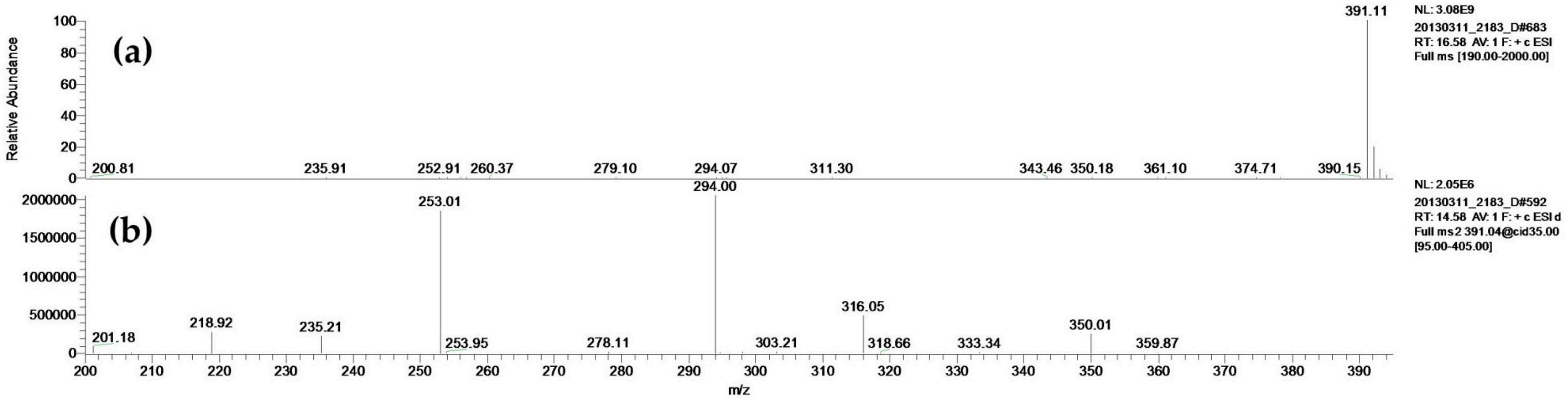

Figure S13. (**a**) Low resolution MS (positive ion mode) of laucysteinamide A (**1**). (**b**) MS/MS (positive ion mode) spectra of **1**.

References

1. Gu, L. C.; Wang, B.; Kulkarni, A.; Gehret, J. J.; Lloyd, K. R.; Gerwick, L.; Gerwick, W. H.; Wipf, P.; Hakansson, K.; Smith, J. L.; Sherman, D. H., Polyketide Decarboxylative Chain Termination Preceded by O-Sulfonation in Curacin A Biosynthesis. *J Am Chem Soc* **2009,** 131, (44), 16033-16035.
2. Chang, Z. X.; Sitachitta, N.; Rossi, J. V.; Roberts, M. A.; Flatt, P. M.; Jia, J. Y.; Sherman, D. H.; Gerwick, W. H., Biosynthetic pathway and gene cluster analysis of curacin A, an antitubulin natural product from the tropical marine cyanobacterium Lyngbya majuscula. *J Nat Prod* **2004,** 67, (8), 1356-1367.
